# Social and demographic drivers of trend and seasonality in elective abortions in Denmark

**DOI:** 10.1186/s12884-017-1397-2

**Published:** 2017-07-04

**Authors:** Tim A. Bruckner, Laust H. Mortensen, Ralph A. Catalano

**Affiliations:** 1 Public Health, University of California, Irvine, 635 E. Peltason Dr, Irvine, CA 92697-7075 USA; 2 Department of Social Medicine, and Statistics Denmark, University of Copenhagen, Øster Farimagsgade 5, Postboks 2099, 1014 København K, Denmark; 3 Public Health, University of California, Berkeley, 50 University Hall, Berkeley, CA 94720 USA

**Keywords:** Elective abortion, Trend, Seasonality, Denmark, Time series

## Abstract

**Background:**

Elective abortions show a secular decline in high income countries. That general pattern, however, may mask meaningful differences—and a potentially rising trend—among age, income, and other racial/ethnic groups. We explore these differences in Denmark, a high-income, low-fertility country with excellent data on terminations and births.

**Methods:**

We examined monthly elective abortions (*n* = 225,287) from 1995 to 2009, by maternal age, parity, income level and mother’s country of origin. We applied time-series methods to live births as well as spontaneous and elective abortions to approximate the denominator of pregnancies at risk of elective abortion. We used linear regression methods to identify trend and seasonal patterns.

**Results:**

Despite an overall declining trend, teenage women show a rising proportion of pregnancies that end in an elective termination (56% to 67%, 1995 to 2009). Non-Western immigrant women also show a slight increase in incidence. Heightened economic disadvantage among non-Western immigrant women does not account for this rise. Elective abortions also show a sustained “summer peak” in June, July and August. Low-income women show the most pronounced summer peak.

**Conclusions:**

Identification of the causes of the increase over time in elective abortion among young women, and separately among non-Western immigrant women, represents key areas of further inquiry. The unexpected increase over time in elective abortions among teens and non-Western immigrants in Denmark may signal important social and cultural impediments to contraception. The summer peak in abortions among low-income women, moreover, conflicts with the conventional assumption that the social and demographic composition of mothers who electively end their pregnancy remains stable within a calendar year.

## Background

In high income countries, elective abortions have shown a secular decline [1, 2]. Denmark, for instance, shows an over 60% reduction, from 1974 to 1995, in the count of elective abortions [2]. Researchers speculate that this decline arises from two main factors: a reduction in the desired number of children and the increasing availability and acceptance of contraception [2]. The overall decline in elective abortion, however, may mask important variation across demographic subgroups [3–6].

We define the incidence of elective abortion as the probability that a pregnancy will terminate in an elective abortion. We use the estimated number of pregnancies as the denominator of the incidence measure. Previous literature in Denmark indicates that immigrant women show a higher odds of elective abortion than do Danish-born women [4]. To the extent that demographic groups with a high incidence of elective abortions signal a lack of knowledge of, or access to, contraceptive options, such information may assist with targeting relevant resources. In addition, identification of socio-demographic differences in temporal patterns of abortion may improve understanding of the determinants of fertility. For instance, relatively fewer elective abortions among older women may indicate that women are increasingly delaying childbearing until older ages.

Elective abortions, moreover, may exhibit patterns other than trend. Prior research reports seasonality in countries such as the U.S. and Australia [7–9]. The Authors of one U.S. study find a summer peak in the count of elective abortions among young women [9]. This research, however, is limited in that it does not take into account the overall increase in the total number of pregnancies at risk of elective termination. Failure to account for seasonal variation in conceptions allows for the possibility that abortions rise in the summer merely due to an increase in conceptions in the spring. This circumstance cannot inform the issue of whether the true “risk” of an elective termination, among current conceptions, varies across seasons of the year.

Describing trends, cycles, and other patterns in elective abortions can contribute to reproductive health in two ways. First, these patterns may suggest environmental or social precursors of termination—especially among sensitive age, income, or parity subgroups [10]. Second, life-course research increasingly reports temporal patterning in clinically important birth outcomes (e.g., preterm parturition) and a relation between month of birth and health over the life course [11, 12]. Much of this work assumes a constant rate of terminations across calendar months. Elective abortions, however, show strong temporal variation. Describing this variation, and any patterns in it, may better illuminate which subgroups of pregnancies (e.g., those among high-income groups) progress to a live birth. Such variation, if patterned, could improve predictions of health in cohorts that survive to birth.

We set out to describe trend and seasonal cycles in elective abortion in Denmark from 1995 to 2009. We use Denmark given its high quality of data on pregnancy termination [13, 14]. Denmark has liberal laws on pregnancy termination, which is performed upon maternal request until the end of the 12th week of pregnancy. Elective termination is free of cost [2]. In addition, Denmark exhibits a total fertility rate below replacement levels [15]. This circumstance mirrors the majority of high income countries. As with previous research, we explore patterns in elective abortion across maternal age, parity, income level and foreign-born status.

## Methods

### Variables and data

We received the following IRB approvals for this project: UC Irvine # 2014–1386 and Danish Data Protection Agency Protocol # 2013–41-2399. We obtained permission to access these non-public data from the Danish Data Protection Agency under this protocol number. We retrieved information on elective abortions and other pregnancy outcomes from Denmark using the National Patient Register and the Medical Birth Register [16, 17]. Denmark’s National Patient Register (NPR) has been described as comprehensive with respect to information on in- and out-patient somatic visits and the ability to link individual data to other health and non-health registries [13]. The NPR includes a unique personal identification number linked to visits, procedures, or diagnoses relating to pregnancy including positive pregnancy tests, receipt of prenatal care, screening for anomalies, and surgeries and deliveries. Beginning in 1995, the NPR contains all outpatient activities and emergency room contacts. This year serves as the start date of our analysis. Our dataset continues until 2009, the last year of full high-quality data available to us.

Denmark permits all women in Denmark aged 18 or above to receive an elective abortion, free of charge, upon maternal request up until the end of the 12th completed week of gestation (Act No. 350, 1973). If the woman is less than 18 years of age, she requires parental consent. We coded elective non-clinically indicated abortion (ICD-10 code: O04) as the principal outcome variable. The NPR also contains information on spontaneous loss, which comprises over 11% of all detected pregnancies in Denmark over the test period. We retrieved counts of spontaneous pregnancy loss (ICD-10 codes: O03, P95), as well as counts of live births, to construct a denominator of the cohort of pregnancies at risk of elective abortion (described further below). We used the exact date of visit associated with these procedure and diagnostic codes to assign the month and year of the event.

The NPR and Medical Patient Register include information on all pregnancies in Denmark that were noted in a clinical setting (e.g., prenatal care visit, positive pregnancy test) as well as elective abortion procedures in Denmark (for 1995 and all subsequent years). These registers include both private and public clinics from all available facilities. Given that elective abortions are free of charge in Denmark and not stigmatized politically, these registers are believed to have almost complete coverage of elective abortions in the country, across all sociodemographic features.

The NPR allows linkage of maternal age, parity, income, and foreign-born information to all pregnancies in Denmark. We focused our exploration on subgroups across these four variables given their documented relevance to reproductive health in Denmark and elsewhere. We classified maternal age into four categories that designate distinct groups in terms of fertility behaviors: <20 years, 20 to 24 years, 25 to 34 years, and 35 to 44 years. We designated three parity groups: nulliparous, primiparous, and parity greater than one. Registers on personal income permitted us to classify the mother’s family income at the time of her pregnancy, which we categorized by income quintile [18]. We classified women’s foreign-born status into three groups: born in Denmark, born outside of Denmark in a Western country (i.e., Andorra, Australia, Canada, Iceland, Lichtenstein, Monaco, New Zealand, Norway, San Marino, Switzerland, USA, the Vatican State, and The European Union member states except Denmark), and in a non-Western country (all other countries). This foreign-born classification coheres with prior research [4]. Migrants from non-Western countries that contribute to the majority of births come from the former Yugoslavia, Pakistan, Turkey, Somalia, and Lebanon [19].

### Analysis

A key “driver” of monthly incidence in elective abortion includes the population of gestations at risk. We know of no population-level data that tracks cohorts of pregnancies from conception through termination. As a surrogate, we used the sum of live births, spontaneous terminations, and elective abortions to approximate the cohort of gestations at risk of elective abortion. We approximated this monthly cohort at risk in two steps. First, we used the monthly count of spontaneous abortions in the same month as elective abortions as “competing risk” gestations that could have been aborted electively. Second, we used autoregressive, integrated, moving average time-series methods [20] to identify the strongest positive correlation of elective abortions with later live births. This analysis discovered the strongest positive correlation at a lag of seven months later. The seven month span coheres with the notion that elective abortions peak in the 6th through 12th week and that the majority of live births occur in the ninth month of gestation (i.e., seven months after the peak in elective abortions). This logic led us to derive an incidence measure of elective abortions at month *t* by specifying at-risk gestations as the sum of elective abortions at month *t*, spontaneous abortions at month *t*, and live births at month *t* + 7. Given the data requirement of live births at month *t* + 7, we restricted the time period of analysis from January 1995 to December 2009. In addition, given that the fraction of births in Denmark that occur in the ninth month of gestation has also remained stable, we used the same incidence calculation for the entire time period.

We first plotted the monthly incidence of elective abortion overall and by demographic subgroup. Second, we employed ordinary-least-squares linear regression analysis to fit a year variable (continuous, from 1 to 15, where 1995 = 1, 1996 = 2, etc.) and 11 binary calendar month variables (i.e., January through November, with December as a referent month). Third, we plotted the calendar month coefficients, with December as the referent coefficient and fixed at 0, to give the reader a sense of calendar and/or seasonal patterns in elective abortions. Fourth, we assessed other functional forms of the year variables, such as quadratic and cubic terms, to determine whether inference about calendar month changed in the presence of non-linear annual trends.

## Results

**Fig. 1:**
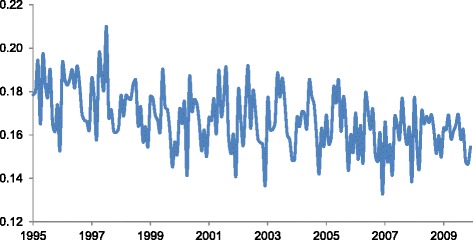
Monthly Iincidence of elective abortions in Denmark (Y-axis) over 180 months (X-axis) spanning January 1995 to December 2009

**Fig. 2:**
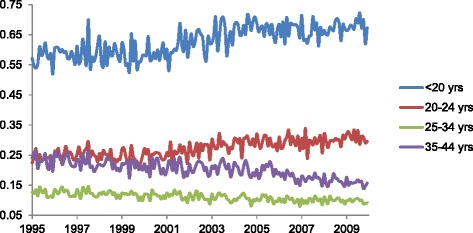
Age-specific plot of monthly incidence of elective abortions in Denmark (Y-axis) over 180 months (X-axis) spanning January 1995 to December 2009

Over the test period, Denmark recorded a total of 225,287 elective abortions. The incidence declines from 1995 to 2009, from 18% to 16% (i.e., proportions of 0.18 to 0.16; see Fig. 1). This decline, however, appears primarily among women aged 35 to 44 years (Fig. 2). By contrast, teenage women show a rising incidence, from 56% to 67%. Elective abortions among women aged 20 to 24 years also shows a gradual increase over time. The year coefficient in the regression results (Table 1) indicates the largest average annual increase (i.e., 0.9%) among women less than 20 years of age.

**Table 1:** Coefficients (standard errors in parentheses) for the constant and year term in linear regression models predicting the incidence of elective abortion. Each row shows results from a separate regression^†^

Elective Abortion Series	Baseline incidence	Yearly change in incidence
Overall	.16 (.002)***	−.0013 (.0001)***
Maternal Age		
< 20 years	.55 (.01)***	.009 (.0005)***
20–24 years	.21 (.005)***	.005 (.0003)***
25–34 years	.11 (.002)***	−.002 (.0001)***
35–44 years	.22 (.004)***	−.005 (.0003)***
Parity		
Nulliparous	.16 (.003)***	−.0003 (.0002)
Primiparous	.09 (.002)***	−.0013 (.0001)***
Parity >1	.26 (.004)***	−.0033 (.0003)***
Income Level		
Lowest quintile	.25 (.006)***	.0003 (.0003)
2nd quintile	.22 (.005)***	−.002 (.0003)***
3rd quintile	.14 (.003)***	.001 (.0002)***
4th quintile	.11 (.003)***	−.0009 (.0002)***
Highest quintile	.17 (.004)***	−.003 (.0002)***
Mother’s Country of Origin		
Denmark	.16 (.002)***	−.0015 (.0001)***
Other Western^‡^	.19 (.008)***	−.0047 (.0005)***
Non-Western	.17 (.005)***	.0029 (.0003)***

^†^coefficients for calendar month not shown; available upon request

^‡^Includes the following countries: Andorra, Australia, Canada, Iceland, Lichtenstein, Monaco, New Zealand, Norway, San Marino, Switzerland, USA, the Vatican State, and The European Union member states except Denmark

***p < .0001

**Fig. 3:**
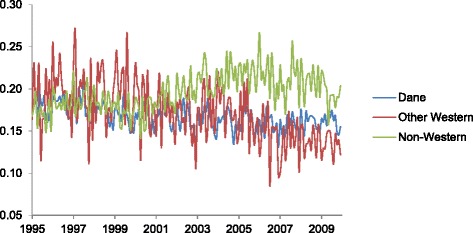
Mother’s country of origin plot of monthly incidence of elective abortions in Denmark (Y-axis) over 180 months (X-axis) spanning January 1995 to December 2009

Of all parity groups, women of parity greater than one show the steepest reduction in elective abortions, with an average annual decline of 0.3% (Table 1). This reduction coheres with the notion that women older than 35 years, with advanced parity, account for a substantial portion of the overall decline. Among income groups, the wealthiest group shows the steepest annual decline, at an average of 0.3% decline per year (Table 1). By contrast, the incidence of elective abortion among the lowest income quintile shows no annual trend (Table 1). Among women of Danish and Western country origin, incidence declines gradually over the test period (Fig. 3 and Table 1). Women of non-Western origin, however, show a distinct rising trend. The overall incidence of elective abortion among women of non-Western origin, moreover, exceeds that of the other country of origin groups for much of the 2000s.

**Fig. 4:**
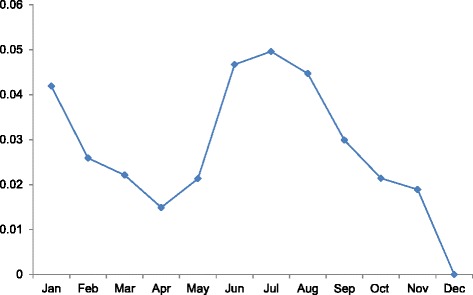
Calendar month coefficients for the overall incidence of elective abortions^†^. ^†^December serves as the referent month, with coefficient fixed at 0

**Fig. 5:**
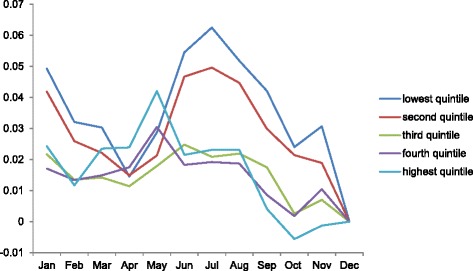
Income-specific, calendar month coefficients for the incidence of elective abortions. December serves as the referent month, with coefficient fixed at 0

**Fig. 6:**
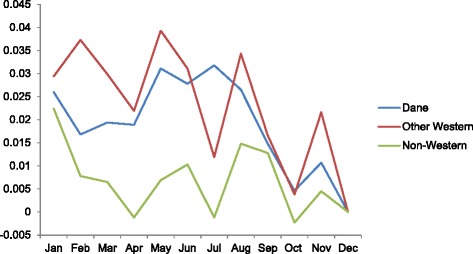
Mother’s country of origin calendar month coefficients for the incidence of elective abortions. December serves as the referent month, with coefficient fixed at 0

Inspection of the calendar month coefficients for elective abortions reveals a rise in Januaries and a second, more sustained peak in June, July and August (Fig. 4). Examination of calendar month patterns among subgroups supports this general summer peak (Figs. 5 and 6). December shows the nadir for virtually all subgroups. One of the most pronounced summer peaks in elective abortions occurs among low-income women (Fig. 5). For women in the lowest and 2nd lowest income quintile, elective abortions exhibit their three highest values in the months of June, July, and August. By contrast, women in the three highest income quintiles show elevated incidence in Januaries and Mays but no peak in the summer. In addition, among country-of-origin groups, only Danish-born mothers exhibit a distinct summer peak (Fig. 6; *p* < .001 for comparison of summer incidence among only Danish-born vs. other two groups). We find no discernable seasonal pattern among women of non-Western origin.

Elective abortions may exhibit non-linear trend over time such that the slope in the 1990s may differ from that in the 2000s (e.g., < 20 age group in Fig. 2). Previous work, for instance, indicates a relative stabilizing of elective abortions from 2003 to 2008 [21]. We assessed this possibility by inserting quadratic and cubic terms for year (i.e., year-squared and year-cubed) in all regressions and identifying whether the coefficients for these terms deviated from the null (*p* < .05). Whereas we find no deviation from linear trend in the overall incidence (Fig. 1), we find in several subgroup analyses that the slope of decline (or increase) in elective abortions varies from 1995 to 2009 (available upon request). This observation indicates that the year coefficients in Table 1 represent the average slope over the entire period but may not describe shorter time spans. In addition, we assessed whether inclusion of non-linear terms for year (e.g., cubic spline, quadratic and cubic terms) altered the coefficients for the month variables shown in Figs. 4 through 6. After inclusion of these terms, all month coefficients remained essentially unchanged from their original tests (available upon request). This robustness check supports the existence of a summer peak in elective abortions that does not arise from confounding by non-linear annual trends.

### Exploration

The rising trend of elective abortions among non-Western women compelled us to explore whether rising economic disadvantage in this group accounts for the increase over time in abortion services. Failure to find stable employment among these immigrants, especially leading up to and including the Great Recession (2008–2009), may reduce strength of social networks and increase the risk of poverty. These sequelae of economic disadvantage, researchers argue, may move women outside of routine pathways to seeking health care and therefore affect their decision to seek abortion [4, 22]. To examine this possibility, we retrieved time series data for non-Western immigrant women with family income in the first or second-lowest income quintile. We reasoned that any rise over time in economic insecurity, which could elicit a subsequent rise in elective abortions, would disproportionately affect these lower-income groups. Regression results indicate no trend in this time series (coef. = .0007, SE = .0005, *p* = 0.15), which suggests that a hypothesized rise in economic insecurity does not account for the rising incidence of elective abortion among non-Western immigrants.

## Discussion

We set out to examine, in a low-fertility, high income context, which socio-demographic subgroups of women account for the secular decline in the incidence of elective abortion. Results from Denmark indicate that women over 25 years of age show a decline from 1995 to 2009 in elective abortion. By contrast, women younger than 25 years show an increase. Immigrant women from non-Western countries exhibit a similar rise in elective abortions over time. This rise among non-Western immigrants does not appear to be explained by increases in economic disadvantage. Identification of the causes of the increase over time in elective abortions among young women, and separately among non-Western immigrant women, represents key areas of further inquiry.

We also explored seasonal patterns of abortion given increasing interest in seasonal antecedents of fertility and cohort health [11, 23, 24]. We find a distinct peak in June, July and August. This summer peak occurs only among women in the two lowest income quintiles. Increases in sexual activity among lower income women during the late spring and early summer cannot fully account for this seasonal peak. Any such increase in coital frequency that raises elective abortions at month *t* would also increase the number of live births at month *t* + 7 and spontaneous abortions at month *t*, which we already capture as the “at-risk” population when deriving incidence. It remains possible, however, that most “excess” conceptions in the spring—among low-income but not high-income women— end in elective abortions in the summer months. Such an explanation presumes that the higher coital frequency in the spring of “high likelihood of abortion” pregnancies occurs predominantly among low-income women.

Another notable difference in seasonality involves the absence of a summer peak of abortion risk for non-Western immigrants relative to Danish-born women (Fig. 6). Different knowledge and cultural attitudes about contraception may play a role. A partner’s negative attitude toward contraception, especially among non-Western immigrant women, may feature prominently in this groups’ higher incidence of abortion across non-summer months [6].

Strengths of our analysis include the use of high-quality data on elective and spontaneous abortions, as well as live births, linked to other patient registers with age, income, parity and country of origin information. Consistency of data collection protocols across time also permits comparability of abortion data over the 15 year period. In addition, the use of time-series methods to empirically derive the denominator of gestations at risk of elective abortions represents an improvement upon previous studies that attempt to control for seasonality or trend in birth rates.

Limitations include that we lacked access to termination and live birth data across other high-income countries. We cannot know whether our age, income, and immigrant-specific results also occur in other high-income, low-fertility countries. We encourage replication efforts to determine the external validity of our results. Lack of reliable annual data in age- and subgroup-specific population counts also prevented us from exploring the number of elective abortions as a function of the total population of women at risk.

Our time-series analysis allows for inference at the aggregate level. The nature of the data did not permit statistical control for multiple individual-level variables simultaneously. For this reason, the reader should interpret our coefficients in Table 1 as reflecting the level, and yearly change in incidence, of elective abortion among groups (rather than of specific individuals). In addition, we do not have survey data on coital frequency or the reasons women sought elective abortions. Next, we observe a local peak in elective abortions in January, but have no post hoc speculation as to its causes. We welcome further investigation into this January peak. Finally, using terminations and live births to approximate the true population of pregnancies at risk of elective abortion likely underestimates our incidence measures. We, however, know of no publicly available dataset which would permit estimates more accurate than ours.

## Conclusions

Our analyses make two important contributions. First, a rising trend in elective abortion among young and non-Western immigrant women holds practical implications for health services. Despite the universal availability and free cost of health care in Denmark, these women in particular may face social, cultural, or structural impediments to contraceptive services [2–4]. This circumstance may characterize other high income countries with a generally declining fertility and abortion rate.

Second, the discovery of a summer peak in the incidence of elective abortion—driven by low-income women—indicates that environmental or social factors that are seasonally patterned may affect the decision of pregnant women to abort. This seasonal pattern in abortions, moreover, conflicts with the conventional assumption that elective abortions remain stable within a calendar year. At least 25% of detected pregnancies in our Danish dataset end in elective or spontaneous termination. For this reason, researchers concerned with the influence of season of birth on infant health [24], and health over the life course [11, 12], may want to account for such month-to-month variation in the composition of cohorts that progress to term.

## Abbreviations

ICD-10: International Classification of Diseases, Tenth Revision
NPR: National Patient Register
